# *Dendrobium officinale* Polysaccharide Relieves the DSS-Induced Chronic Colitis in C57BL/6J Mice and Regulates Colonic Microflora Structure

**DOI:** 10.3390/metabo15110708

**Published:** 2025-10-30

**Authors:** Yangyu Ma, Jingrui Li, Xianling Yuan, Wenyang Tao, Wanyi Zhou, Jianrong Xing, Ying Yang, Haihua Zhang

**Affiliations:** 1 National Key Laboratory for Development and Utilization of Forest Food Resources, The College of Food and Health, Zhejiang A&F University, Hangzhou 311300, China; 2023613022023@stu.zafu.edu.cn; 2 Institute of Food Science, Zhejiang Academy of Agricultural Sciences, Hangzhou 310021, China; 3 Institute of Functional Food, Zhejiang Dongri Food Science Research Center, Wenzhou 325006, China

**Keywords:** *Dendrobium officinale*, polysaccharides, chronic colitis, gut microbiota, metabolic pathways

## Abstract

**Background/Objectives**: Chronic colitis presents a growing global health burden with rising incidence. This study investigated the ameliorative effect of *Dendrobium officinale* polysaccharide (DOP) against dextran sulfate sodium (DSS)-induced chronic colitis in mice, specifically examining its dual modulation of gut microbiota and metabolic pathways. **Methods**: DOP was extracted and purified from *Dendrobium officinale* stems and leaves. A chronic colitis model was established in male C57BL/6J mice via DSS induction. Eighty-four mice were randomized into seven groups: control, model, low/high-dose leaf-DOP, low/high-dose stem-DOP, and sulfasalazine positive control. We assessed body weight, disease activity index (DAI), colon length, splenic/thymic indices, inflammatory cytokines, and histopathology (Hematoxylin and Eosin/Alcian blue staining), with tight junction protein and tumor necrosis factor-alpha (TNF-α) expression quantified via immunofluorescence. 16S rRNA sequencing and untargeted metabolomics evaluated microbial and metabolic shifts. **Results**: DOP significantly attenuated colitis severity, restored colon histoarchitecture, elevated goblet cell counts, upregulated zonula occludens-1 (ZO-1) and occludin expression, and suppressed TNF-α. Crucially, DOP remodeled dysbiosis by enriching beneficial taxa (e.g., *Candidatus_Saccharimonas*, *Lachnoclostridium*) while reducing pathogens (*Mucispirillum*). Metabolomics further elucidated DOP-mediated regulation of purine and nicotinate/nicotinamide metabolism—pathways mechanistically linked to its anti-inflammatory and barrier-repair effects. **Conclusions**: DOP effectively alleviates symptoms of DSS-induced chronic colitis in mice, protects intestinal barrier integrity, and achieves therapeutic potential through simultaneous regulation of the gut microbiome and metabolome.

## 1. Introduction

Chronic colitis, a complex inflammatory bowel disease characterized by a protracted clinical course and high relapse rates, significantly compromises patients’ quality of life [1]. Current pharmacological therapies for colitis include sulfasalazine (SASP), corticosteroids, and immunosuppressants. These agents ameliorate inflammation of the intestinal tissue and alleviate associated clinical manifestations [2]. SASP serves as a targeted prodrug: following oral administration, it remains chemically stable throughout the upper gastrointestinal tract until colonic bacterial azoreductases cleave its azo bond. This site-specific activation enables potent anti-inflammatory effects in the colon, including suppression of inflammatory mediators and scavenging of oxygen-free radicals [3]. However, long-term SASP administration carries risks of significant adverse effects [4]. Consequently, developing safer and more effective alternative therapies represents a critical research objective. Investigation of disease pathology and prevention strategies commonly employs the dextran sulfate sodium (DSS)-induced chronic colitis murine model [5].

Mounting evidence establishes a close relationship between the pathogenesis of chronic colitis and intestinal microbiome dynamics [6]. Gut microbiota and their metabolites significantly regulate host homeostasis through nutrient processing, immune defense, energy homeostasis maintenance, and immune system development [7]. Crucially, patients with chronic colitis exhibited a distinct gut microbial composition relative to healthy subjects, characterized by a depletion of beneficial bacteria (e.g., *Lactobacillaceae*) and an enrichment of inflammation-associated bacteria (e.g., *Helicobacter pylori*) [8]. Microbial metabolic reprogramming, fundamentally governing energy–biomolecule interconversions, represents a core pathogenic mechanism in chronic colitis development. Specifically, dominant gut flora produced SCFAs as primary metabolites via ^13^C-glucose fermentation, thereby indirectly regulating energy metabolism, repairing intestinal barriers, and maintaining mucosal homeostasis [9]. Consequently, delineating chronic colitis-associated microbiota–metabolite interactions that fuel inflammatory and repair processes is essential to elucidate disease etiology and advance novel therapies [10].

As a traditional Chinese herbal medicine, *Dendrobium officinale* (DO) exhibits various pharmacological effects such as clearing heat and generating fluids, nourishing Yin, and nourishing the stomach [11]. In recent years, *Dendrobium officinale* polysaccharide (DOP) has garnered significant scientific interest due to its distinctive bioactive properties. DOP exhibits diverse pharmacological activities, including antioxidant, anti-inflammatory, and antitumor effects [12]. It also modulates gut microbiota composition and enhances intestinal microecological homeostasis, thereby alleviating intestinal inflammation [13]. Although polysaccharides are present in both *Dendrobium officinale* leaves and stems, their quantities notably differ. Crucially, it remains unresolved that polysaccharides from different parts of DO (leaves vs. stems) have differential inhibitory effects on chronic colitis through gut microbiota restructuring and metabolite regulation.

In this study, DSS mimicked the pathological changes in human chronic colitis by disrupting the intestinal mucosal barrier and triggering immune responses and inflammatory processes [14]. This study evaluated the preventive treatment effects of polysaccharides from *Dendrobium officinale* leaf sheaths (DOPY) and stems (DOPJ) on chronic colitis, utilizing clinical disease activity indices and histopathological assessments. Through comparative analysis, we identified the distinct effects of DOPY versus DOPJ on gut microbiota composition (16S rRNA sequencing) and metabolite profiles (untargeted metabolomics), illustrating DOP’s function as a modulator of gut microbiota-metabolite cross-talk. These findings establish a theoretical foundation for elucidating the molecular mechanisms underlying plant-derived polysaccharides in preventing chronic colitis, while facilitating the development and application of DOP-based therapeutics for colitis management.

## 2. Materials and Methods

### 2.1. Isolation and Purification of DOP

Fresh stems and leaves of DO were crushed and soaked in distilled water at ratios of 25 mL/g for stems and 5 mL/g for leaves, followed by extraction at 70 °C for 40 min. After cooling and filtration through double-layered gauze, the filtrate was centrifuged (4800 rpm, 12 min), and the supernatant was passed through a 2000-mesh filter cloth. Ethanol precipitation (5 volumes of 95% ethanol, 24 h) yielded crude polysaccharides, which were collected via 8000-mesh filtration and dried. The dried polysaccharides were redissolved in deionized water (1:50 *w*/*v*) and homogenized under continuous magnetic stirring (75 °C, 300 rpm). After sequential dialysis (MWCO 8–14 kDa) and lyophilization (−50 °C, 0.1 mbar, 72 h) to obtain the two polysaccharide fractions (DOPY and DOPJ).

Structural analysis revealed that mannose and glucose constituted the primary monosaccharides (>90% combined content) in both DOPY and DOPJ, with minor contributions from galactose and arabinose. The molar ratios were 62.34:3.48:1.57:1 (mannose: glucose: galactose: arabinose) for DOPY and 435.07:275.93:2.29:1 for DOPJ. Molecular weight characterization by high-performance gel permeation chromatography (HPGPC) indicated two fractions for DOPY (146.49 and 1.25 kDa) and three for DOPJ (1699.28, 12.98, and 1.33 kDa). Fourier Transform Infrared Spectroscopy (FT-IR) spectral analysis confirmed characteristic polysaccharide absorption bands at 3410 cm^−1^ (O–H stretching), 2920 cm^−1^ (C–H stretching), 1627 cm^−1^ (C=O stretching), and 1025 cm^−1^ (β-Pyranose stretching). Structural data collectively demonstrate that DOPY and DOPJ adopt β-pyranose configurations. These results are consistent with previous studies [15].

### 2.2. Animals

Male C57BL/6 mice (*n* = 84; age 7 weeks, weight 20 ± 2 g) were procured from Hangzhou Paisa Biotechnology (Hangzhou City, Zhejiang Province, China). The mice were maintained in SPF facilities under strictly controlled conditions: a temperature of 24 ± 2 °C, a relative humidity of 45 ± 10%, and a standardized 12 h light/12 h dark cycle. Unrestricted access to pelleted feed and sterile water was ensured.

### 2.3. DSS-Induced Chronic Colitis Model

**Figure 1:**
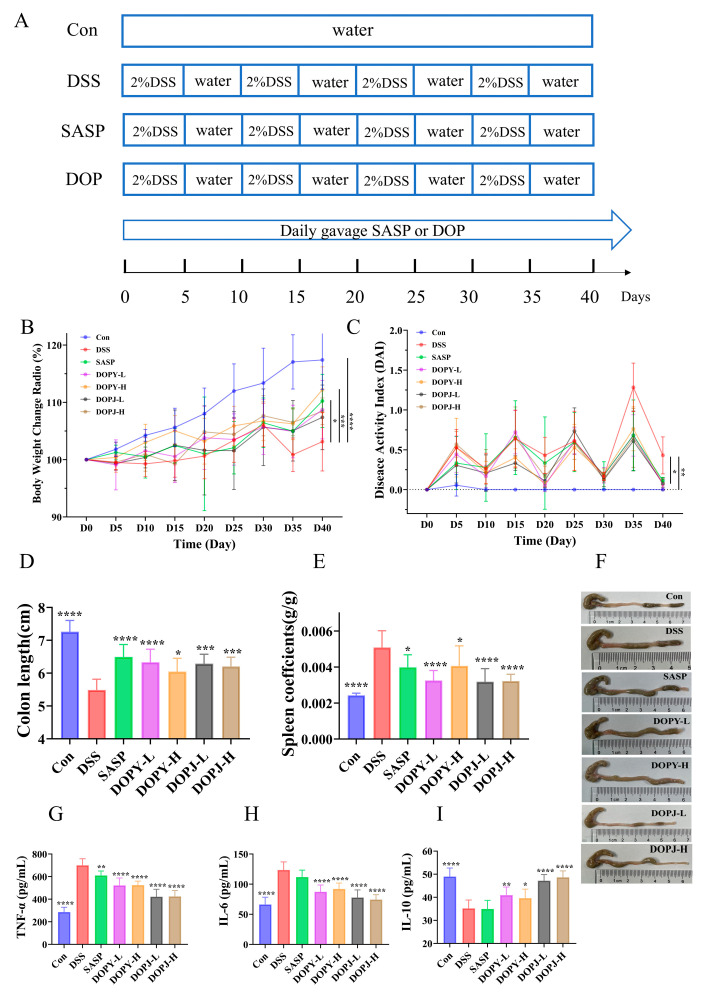
DOP ameliorated DSS-induced chronic colitis in mice (*n* = 12). (**A**) Chronic colitis mouse model experimental design; (**B**) Body weight dynamics in DSS-Induced colitis; (**C**) DAI values; (**D**) Colon length; (**E**) Spleen index scores; (**F**) Photographs of the colon; (**G**) TNF-α levels in the colon tissues; (**H**) IL-6 levels in the colon tissues; (**I**) IL-10 levels in the colon tissues. Data are expressed as mean ± SD. * *p* < 0.05, ** *p* < 0.01, *** *p* < 0.001, and **** *p* < 0.0001 compared to the DSS group (the model control group).

After one week of acclimatization, the mice were randomized into seven groups (*n* = 12): (1) NC (normal control: distilled water); (2) DSS (model: DSS alone); (3) SASP (positive drug control: 450 mg/kg/day); (4) DOPY-L (low-dose polysaccharides from *Dendrobium officinale* leaves, 50 mg/kg/day); (5) DOPY-H (high-dose polysaccharides from *Dendrobium officinale* leaves, 100 mg/kg/day); (6) DOPJ-L (low-dose polysaccharides from *Dendrobium officinale* stems, 100 mg/kg/day); (7) DOPJ-H (high-dose polysaccharides from *Dendrobium officinale* stems, 200 mg/kg/day). In the chronic colitis model, mice in the normal control group received a standard diet and water without intervention. For the other groups, chronic colitis was induced by cyclic administration of 2% DSS (MP Biomedicals, Santa Ana, CA, USA): Mice were maintained on a standard diet while receiving 2% DSS solution ad libitum as drinking water for 5 days, with fresh DSS solution prepared every 48 h, followed by 5 days of sterile water—constituting one 10-day cycle. This cycle was repeated four times to establish the chronic colitis model (Figure 1A) [16]. Importantly, during the DSS exposure phases across all cycles, all groups received their respective gavage treatments concurrently with DSS administration. This included daily oral gavage for polysaccharide/drug solutions or sterile water, initiated from Day 1 of the first DSS cycle. Specifically, mice were treated with DSS water throughout the experimental period. Beginning at cycle 1 (Day 1), DOP-treated groups received polysaccharide solutions via oral gavage (0.1 mL/10 g body weight at respective doses), while the SASP group received sulfasalazine. Both disease-model controls and healthy controls received sterile water at identical volumes, administered daily via oral gavage.

At the study endpoint (day 40), all mice (*n* = 84) underwent a 12 h fast with ad libitum access to water. Animals were humanely euthanized under isoflurane (Hangzhou, China) anesthesia via cardiac puncture. Serum isolation was performed by centrifugation at 3000× *g* (15 min, 4 °C). Immune organs (spleen) were weighed on analytical balances (±0.1 mg). Ileal and colonic luminal contents were aseptically collected for microbial profiling. Colon tissues were collected and stored at −80 °C for subsequent analysis.

### 2.4. Disease Activity Index (DAI) Measurement

Mice underwent daily body weight measurements at consistent times throughout the study. Stool properties were documented, and fecal occult blood tests assessed rectal bleeding. These parameters were assessed and averaged into the DAI according to the scoring instructions in Table S1 [17] (where body weight loss, stool consistency, and rectal bleeding are classified on defined scales), to provide a standardized preliminary assessment of model validation and therapeutic effects. The total DAI score was derived by calculating the mean of the three individual scores with the following formula:

DAI = (body weight loss + stool consistency + fecal blood)/3.(1)

### 2.5. Hematoxylin and Eosin (H&E), Alcian Blue, and Immunohistochemical Staining

Following treatment completion, the distal colonic segments were harvested and subjected to 4% paraformaldehyde fixation, paraffin embedding, and sectioning at a thickness of 4 μm. These sections underwent H&E and Alcian blue staining for subsequent histopathological assessment via light microscopy [18]. Then, the number of cup cells was counted using ImageJ software (version 1.8.0; National Institutes of Health, Bethesda, MD, USA).

Dewaxed and rehydrated colonic sections were phosphate-buffered saline (PBS, Beijing, China)-rinsed, blocked in 10% goat serum, and incubated serially with primary (4 °C overnight) and secondary antibodies (37 °C, 30 min). Counterstaining with diaminobenzidine/hematoxylin (Kalamazoo, MI, USA) facilitated microscopic evaluation and documentation of ZO-1, occludin, and tumor necrosis factor-alpha (TNF-α) localization.

### 2.6. Evaluation of Inflammation

Freshly excised intestinal tissues were homogenized in a 1:9 (*w*/*v*) PBS mixture using mechanical disruption equipment [19]. Subsequently, TNF-α, Interleukin-6 (IL-6), and Interleukin-10 (IL-10) levels were quantified following enzyme-linked immunosorbent assay (ELISA) kits (Hangzhou Lianke Biotechnology Co., Ltd., Hangzhou, China) according to the instructions.

### 2.7. Colonic Microflora Analysis

Fecal samples were collected from each of the mouse groups. Total DNA was extracted from stool specimens according to the instructions. Qualified DNA extracts underwent amplification of the bacterial 16S rRNA V3-V4 gene using universal primers 338F (5′-ACTCCTACGGGGAGGCAGCA-3′) and 806R (5′-GGACTACHVGGGGTWTCTAAT-3′). Following PCR amplification, amplicons were purified and quantified. Both forward and reverse primers contained unique sequences for each sample to enable multiplex sequencing. The Miseq PE300/NovaSeq PE250 platform was used to sequence the obtained libraries [20]. It is important to note that 16S rRNA gene sequencing provides data on the relative abundance of bacterial taxa within a sample, reflecting community composition, but does not measure absolute abundance or total bacterial load.

### 2.8. Non-Targeted Metabolomics Analysis

Exactly 50 mg of murine feces was homogenized in an extraction solution (methanol: water = 4:1, *v*/*v*) spiked with internal standards (e.g., L-2-chlorophenylalanine, 0.02 mg/mL). Samples were ground for 6 min at −10 °C in a cryogenic tissue homogenizer, followed by ultrasound-assisted extraction (5 °C, 40 kHz) for 30 min. Protein precipitates formed at −20 °C (30 min) were centrifuged (4 °C, 13,000× *g*, 15 min). Resultant supernatants underwent LC-MS profiling. Detailed analytical conditions are provided in the Supplementary Materials.

Raw data were processed by Progenesis QI v3.0 software (Nonlinear Dynamics, Newcastle, UK). Then, MS/MS spectra were matched against metabolic databases. Primary reference databases included: HMDB (http://www.hmdb.ca, accessed on 30 July 2025), METLIN (https://metlin.scripps.edu, accessed on 30 July 2025), and an in-house library curated with authentic standards.

### 2.9. Statistical Analysis

Values expressed as mean ± SEM (*n* ≥ 6). Group differences were assessed by one-way analysis of variance (ANOVA) with Tukey’s multiple comparison test using GraphPad Prism 9.0 and MajorGene Cloud (Shangshai, China). Statistical significance required *p* < 0.05.

## 3. Results

### 3.1. DOP Alleviates Colitis Symptoms in Chronic Colitis Mice

Clinical assessment of chronic colitis severity and prognosis relies on key indicators, including weight loss, alterations in food/water intake, and the DAI (stool characteristics, specifically consistency and blood content). DSS-induced mice exhibited significant weight reduction compared to controls (*p* < 0.001) from Day 30, whereas both SASP and DOP interventions attenuated weight loss; however, the effects of SASP (*p* < 0.05) and DOPY-H (*p* < 0.001) were more significant (Figure 1B). Similarly, DAI was significantly elevated in the DSS group, while both SASP and each dose of DOP significantly reduced DAI index (*p* < 0.05, Figure 1C), confirming the protective effect of DOP in ameliorating chronic colitis disease activity. Relative to controls, DSS administration substantially reduced colon length (*p* < 0.0001, Figure 1D). Compared with the model group (*p* < 0.05), the preventive treatment intervention with SASP or DOP significantly reversed this shortening phenomenon, among which SASP and DOPY-L achieved the maximum length recovery (*p* < 0.0001). Notably, in the photographs of the colon shown in Figure 1F, colonic specimens from DSS-treated mice, in addition to being significantly shorter, had edema and congestion pathology, which were significantly improved after the SASP and DOP interventions. This underscores the protective and restorative effects of DOP against DSS-induced intestinal damage. Furthermore, the administration of DOP as an intervention also significantly lowered the splenic index in chronic colitis mice (Figure 1E), indicating its capacity to normalize immune function in DSS-induced colitis by suppressing splenomegaly. Notably, DOPY-L and DOPJ groups demonstrated superior efficacy in attenuating splenic enlargement (*p* < 0.001).

ELISA revealed profound elevation of pro-inflammatory TNF-α and IL-6 alongside reduced anti-inflammatory IL-10 in DSS-group colonic tissues versus controls (*p* < 0.001; Figure 1G–I). Administration of both DOP and SASP attenuated these cytokine alterations, lowering TNF-α and IL-6 while increasing IL-10. In particular, cytokine profiles were markedly restored in DOP groups of mice compared to DSS controls (*p* < 0.05), with DOPJ showing greater potency than SASP and outperforming DOPY.

### 3.2. Interventional Therapy with DOP Attenuates DSS-Induced Colon Tissue Injury

**Figure 2:**
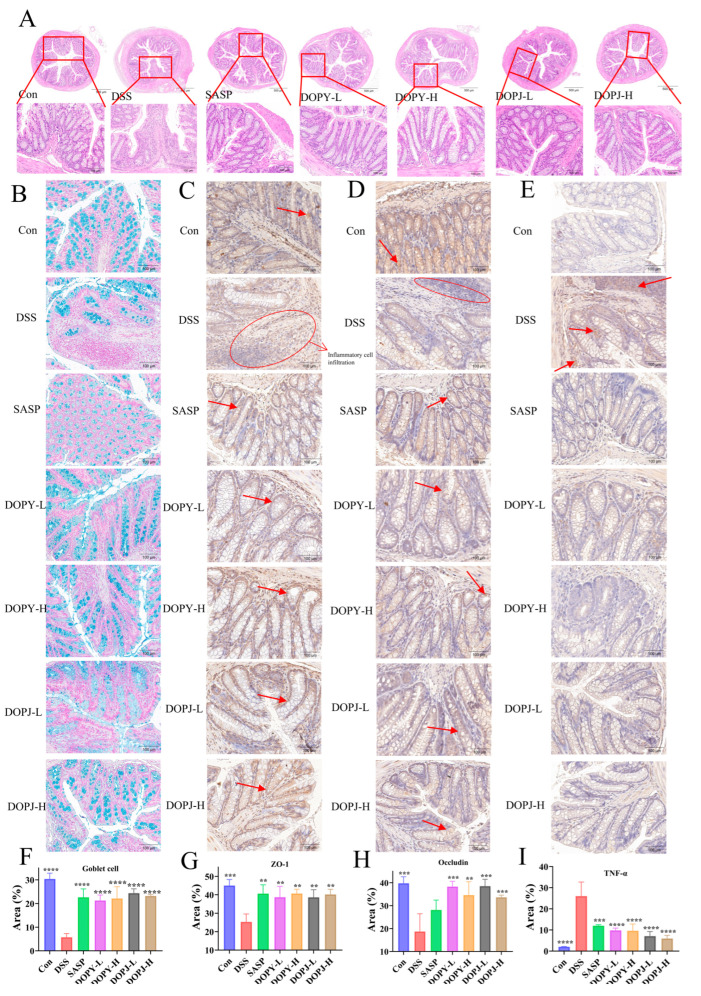
DOP alleviated colonic tissue damage in chronic colitis mice (*n* = 12). (**A**) H&E staining; (**B**) Alcian blue staining; (**C**–**E**) Immunohistochemistry of Zonula occludens-1 (ZO-1), occludin, and TNF-α; (**F**) Relative expression of Alcian blue staining in colonic tissues of goblet cells; (**G**–**I**) Relative expression of immunohistochemistry of ZO-1, occludin, and TNF-α. Data are expressed as mean ± SD. ** *p* < 0.01, *** *p* < 0.001, and **** *p* < 0.0001 compared to the model control group. The arrow indicates the presence of the positive signal.

Colonic histology in control mice demonstrated abundant goblet cells alongside crypts of normal structure and continuous mucosal epithelium. DSS administration, however, led to significant histopathological alterations, including diminished goblet cells, architectural crypt disruption, inflammatory cell infiltration, loss of surface epithelium, and frank mucosal ulceration (Figure 2A). In comparison to the DSS group, the SASP and DOPY groups exhibited greater epithelial integrity and reduced inflammatory infiltration. Figure 2B revealed markedly fewer goblet cells in the DSS group versus the Con group, whereas the SASP and DOP groups exhibited greater goblet cell abundance. This was quantified in Figure 2F, where the SASP and DOP groups showed significantly elevated goblet cell levels compared to the DSS controls. ZO-1 and occludin expression significantly decreased in the DSS group (Figure 2C,D). Conversely, both proteins were upregulated in SASP and DOPY groups, with DOP groups showing significantly increased levels (Figure 2G,H; *p* < 0.01). TNF-α was markedly elevated in DSS-treated mice compared to controls, but decreased substantially after SASP/DOP interventions (Figure 2E,I). This trend in TNF-α levels between colon tissue and serum remained consistent (Figure 1G). These findings demonstrate that DOP exhibited comparable efficacy to SASP—a first-line mucosal inflammation therapy drug—suggesting its potential for clinical translation.

### 3.3. DOP Modulates the Gut Microbiota

**Figure 3:**
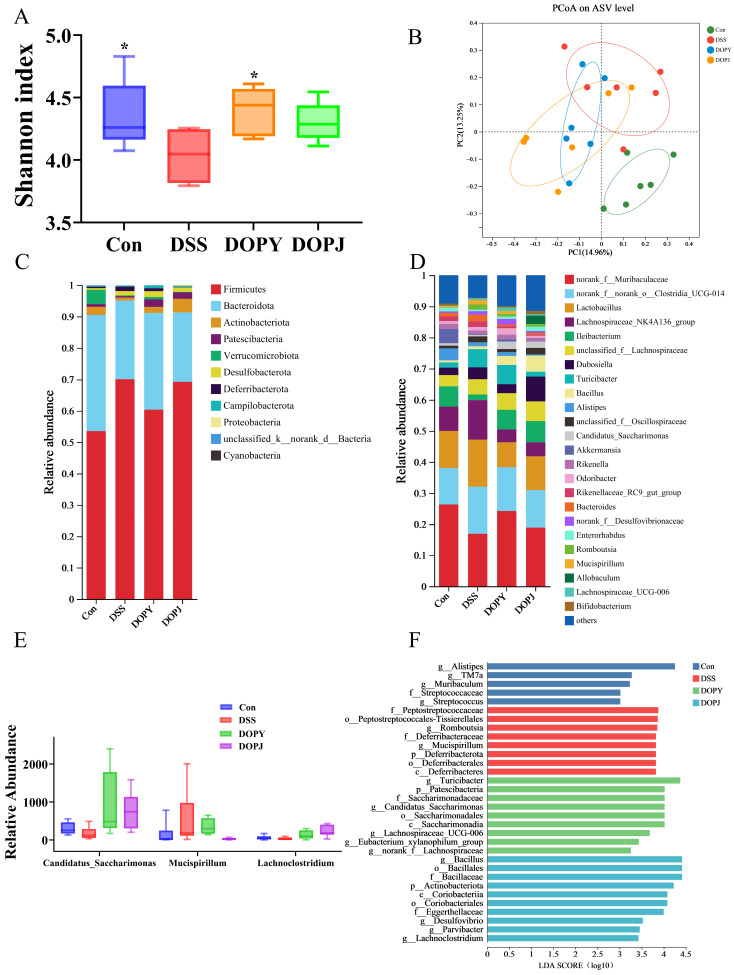
DOP intervention significantly improved the gut microbiota of the mice (*n* = 6). (**A**) Shannon index; (**B**) PCoA analysis; (**C**) community composition at the phylum level; (**D**) community composition at the genus level; (**E**) Relative abundance of Candidatus-Saccharimonas, Mucispirillum, Lachnoclostridium; (**F**) LefSe Bar. Data are expressed as mean ± SD. * *p* < 0.05, compared to the DSS group.

Based on the above findings, the low-dose leaf and stem polysaccharide groups, showing optimal ameliorative indices, were selected for 16S rRNA sequencing. As illustrated in Figure 3A, the Shannon diversity index exhibited a notable reduction in the colitis model compared to the control group, while leaf polysaccharide intervention restored microbial richness.

Beta-diversity assessment revealed distinct clustering patterns among experimental groups (Figure 3B). Polysaccharide-supplemented groups clustered closer to the control group, while distinctly separating from the DSS-induced colitis model, suggesting that polysaccharide intervention promoted restoration of the microbial community.

At the phylum level, the gut microbiota in all experimental groups was dominated by *Firmicutes*, *Bacteroidetes*, *Actinobacteriota*, and *Patescibacteria* (Figure 3C). Notably, the DSS group showed a significant increase in *Firmicutes* relative abundance. At the genus level, *norank_f_Muribaculaceae*, *norank_f_norank_o_Clostridia_UCG-014*, *Lactobacillus*, and *Lachnospiraceae_NK4A136_*group were the most prevalent taxa (Figure 3D). Particularly striking was the substantial rise in *Bacteroides* relative abundance in the DSS group, establishing it as a dominant genus. Oral administration of DOPJ effectively restored *Bacteroides* levels to those observed in the Con group. These findings suggest that DSS treatment selectively enhanced *Bacteroides* proliferation, while DOPJ intervention significantly reshaped this microbial profile. This observation is clinically relevant, given the well-established association between elevated *Bacteroides* levels and colitis-associated dysbiosis, implying a potential mechanistic link between *Bacteroides* expansion and DSS-induced intestinal inflammation [21].

Comparative microbial analysis (Figure 3E) revealed reduced relative abundances of *Candidatus_Saccharimonas* and *Lachnoclostridium* in the DSS group compared to the Con group, alongside increased *Mucispirillum* levels. DOP intervention reversed these DSS-induced perturbations, significantly restoring *Candidatus*-*Saccharimonas* and *Lachnoclostridium* while suppressing *Mucispirillum* relative to DSS-only mice. LefSe analysis (Figure 3F) identified taxa with significant differential relative abundances across groups. The higher LDA scores for *Alistipes*, *Turicibacter* (a well-documented anti-inflammatory genus), and *Bacillus* suggest their potential as key discriminators of inter-group microbial community structures.

Collectively, DOP enhanced overall α-diversity (Figure 3A) and induced distinct β-diversity clustering (Figure 3B), indicating broad structural reorganization of microbial communities. These results demonstrate that DOP ameliorates DSS-induced gut dysbiosis through a dual mechanism: enriching beneficial taxa (*Candidatus_Saccharimonas, Lachnoclostridium, Turicibacter, Bacillaceae*) linked to anti-inflammation, barrier protection, and metabolite production, while suppressing conditionally pathogenic genera (*Bacteroides, Mucispirillum*) implicated in colitis exacerbation. This microbial remodeling constitutes a key mechanism underlying DOP’s efficacy in alleviating experimental colitis.

### 3.4. DOP Regulates the Function of Metabolic Pathways in Chronic Colitis Mice

In both ion modes, the metabolic profiles of Con, the low-dose leaf and stem polysaccharide groups, were distinctly different (Figure S1A–F), indicating significant alterations in host metabolic profiles across DSS-induced colitis and DOP intervention in murine models. To confirm reliability, we used a 200-permutation test on the OPLS-DA models. The key was to check if the intercept between Q2 and the *Y*-axis was <0.5. A smaller intercept indicates a more reliable model that is less prone to overfitting. Our results showed no overfitting in either model (Figure S1G–L), confirming the validity of our data.

**Figure 4:**
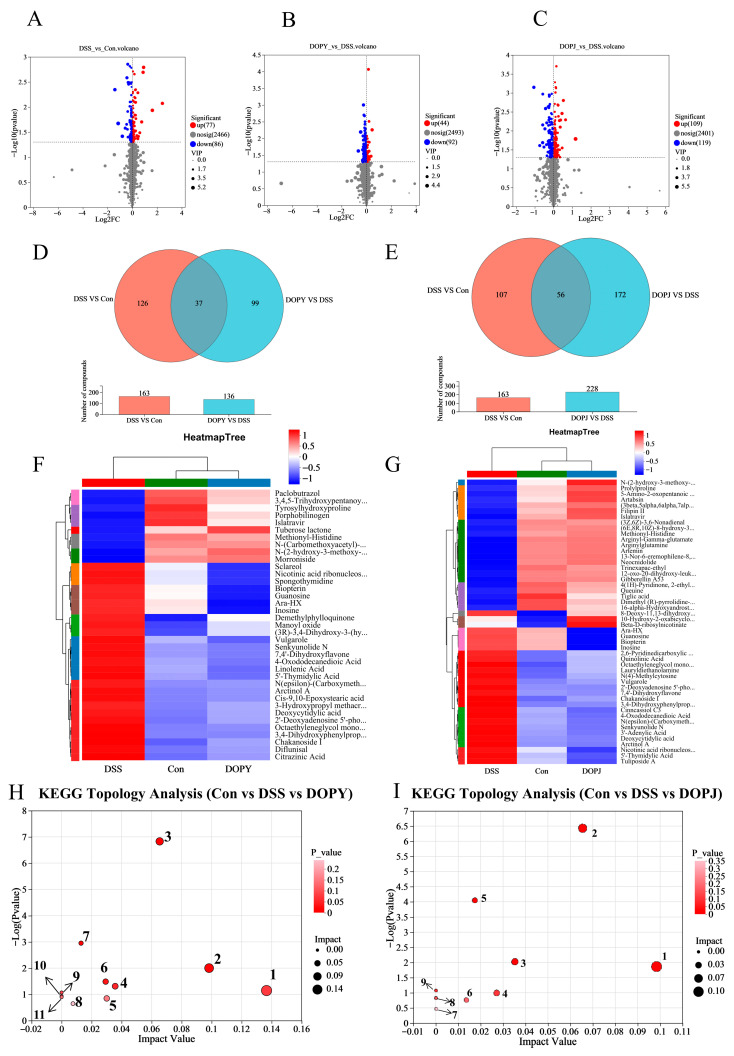
Screening of differential metabolites and associated metabolic pathways is presented. ((**A**): Con vs. DSS; (**B**): DSS vs. DOPY; (**C**): DSS vs. DOPJ) Figure panels depict: volcano plots; ((**D**): Con/DSS/DOPY; (**E**): Con/DSS/DOPJ) Venn diagrams of shared metabolites; ((**F**): Con/DSS/DOPY; (**G**): Con/DSS/DOPJ) abundance heatmaps where color scales (blue → red) indicate expression gradients; ((**H**): Con/DSS/DOPY; (**I**): Con/DSS/DOPJ) Metabolic pathway analysis.

Differential metabolites were screened using dual thresholds: statistical significance (*p* < 0.05, Student’s *t*-test) and orthogonal partial least squares-discriminant analysis (OPLS-DA) variable importance (VIP > 2.0). Metabolomic profiling identified: Con vs. DSS: 163 significantly altered metabolites (77 upregulated, 86 downregulated; Figure 4A). DOPY vs. DSS: 136 differential metabolites (44 upregulated, 92 downregulated; Figure 4B). DOPJ vs. DSS: 228 metabolite perturbations (109 upregulated, 119 downregulated; Figure 4C). Cross-comparative Venn analysis revealed 37 shared metabolites between Con-DSS and DSS-DOPY comparisons (Figure 4D, Table S2), and 56 overlapping metabolites in Con-DSS versus DSS-DOPJ groups (Figure 4E, Table S3). Hierarchical clustering revealed close metabolic proximity between the DOP preventive treatment group and the control group, with a marked separation from the DSS-induced colitis cohorts (Figure 4F,G). This metabolic realignment suggests that DOP has potent regulatory effects in attenuating pathological metabolic dysfunction. Consequently, these conserved differential metabolites may suggest potential therapeutic targets for DOP-mediated intervention in colitis.

Metabolic pathway analysis revealed significant alterations in metabolic pathways induced after DOP protective treatment. For DOPY preventive treatment group, the affected pathways (Figure 4H) included: 1: alpha-Linolenic acid metabolism, 2: Pyrimidine metabolism, 3: Nucleotide metabolism, 4: Cutin, suberine and wax biosynthesis, 5: Ubiquinone and other terpenoid-quinone biosynthesis, 6: Biosynthesis of cofactors, 7: Purine metabolism, 8: Porphyrin metabolism, 9: Biosynthesis of unsaturated fatty acids, 10: Nicotinate and nicotinamide metabolism, 11: Folate biosynthesis. Meanwhile, For DOPJ preventive treatment group, the affected pathways (Figure 4I) namely: 1: Pyrimidine metabolism, 2: Nucleotide metabolism, 3: Nicotinate and nicotinamide metabolism, 4: Pentose phosphate pathway, 5: Purine metabolism, 6: Biosynthesis of cofactors, 7: Biosynthesis of nucleotide sugars, 8: alpha-Linolenic acid metabolism, 9: D-Amino acid metabolism. Notably, during the preventive process against DSS-induced chronic colitis, both DOPY and DOPJ exhibited overlapping metabolic pathways, with purine metabolism emerging as a key pathway. This finding is consistent with our previous results on the effects of DOP in a mouse model of acute colitis [22]. Furthermore, long-term intervention in the chronic colitis mouse model revealed alterations in a broader spectrum of metabolic pathways. These results indicate that DOP intervention can induce metabolic reprogramming in the intestinal cells of mice with chronic colitis.

## 4. Discussion

Clinical assessment of chronic colitis severity and prognosis relies on key indicators, including weight loss, alterations in food/water intake, and the DAI (stool characteristics, including consistency and blood content) [23]. In this study, groups that received preventive intervention with DOPY and DOPJ showed a significant reduction in the DAI score (Figure 1C) compared to the DSS model group, indicating that DOP intervention alleviated the inflammatory response in mice with DSS-induced chronic colitis. It is noteworthy that during the later stage of the experiment (days 35–40), the DOPY-intervention group demonstrated superior improvement in body weight compared to the DOPJ group, showing a dose-dependent trend. Both intervention groups exhibited increased colon length and a reduced splenic index compared to the DSS model group (Figure 1D–F). As a primary immune organ, splenomegaly reflects a state of systemic immune activation during colitis. This process involves massive infiltration of immune cells and excessive cytokine secretion, which has been proven to exacerbate colonic inflammation [16].

The imbalance in the expression of pro- and anti-inflammatory cytokines plays a crucial role in the pathogenesis of chronic colitis [24]. Therefore, intervention strategies capable of modulating the levels of such cytokines are of significant importance. To further investigate the anti-inflammatory effects of DOP in mice with colitis, we analyzed cytokine levels in colonic tissues using ELISA. The results showed that after DOP intervention, the levels of pro-inflammatory cytokines TNF-α and IL-6 were significantly decreased, while the level of the anti-inflammatory cytokine IL-10 was markedly increased in the mouse colon (Figure 1G–I). These findings indicate that DOP possesses potent anti-inflammatory properties, possibly achieved through modulating the expression of inflammatory cytokines to suppress intestinal inflammation.

One of the most important findings of this study is the evidence that low-dose DOP exhibits greater efficacy than high-dose DOP in ameliorating DSS-induced colitis. This non-monotonic dose–response relationship is frequently observed with bioactive immunomodulators, particularly natural products, which are widely recognized for their ability to modulate rather than simply suppress immune activity. Such agents can differentially regulate immune responses depending on both the physiological context and the administered dose [25]. In this study, low-dose DOP may promote beneficial immunomodulation by inducing macrophage polarization toward an anti-inflammatory M2 phenotype, as supported by elevated IL-10 levels in the DOPY-L group. In contrast, high-dose DOP may lead to excessive immune activation, potentially resulting in paradoxical pro-inflammatory effects or receptor desensitization. This is exemplified by the higher TNF-α levels observed in the DOPY-H and DOPJ-H groups compared to their low-dose counterparts, a biphasic pattern consistent with the immunomodulatory dynamics illustrated in Figure 1E. Many studies [26,27] have reported that polysaccharides such as β-glucan can promote the phenotypic polarization of macrophages from M1 to M2, exhibiting anti-inflammatory and pro-repair properties.

The intestinal epithelium maintains intestinal homeostasis and suppresses inflammation through its barrier function [28]. Mucin secretion (primarily governed by goblet cells) and tight junction integrity are critical for this barrier [29]. DSS-induced depletion of goblet cells in murine intestines led to profound mucin deficiency, bacterial translocation, and exacerbated inflammation. In contrast, DOP intervention restored goblet cell populations in colonic tissues. As key tight junction constituents, ZO-1 and occludin serve as definitive markers of epithelial integrity [30]. Moreover, the elevated levels of TNF-α may contribute to destroying the tight junction of intestinal epithelial cells and further aggravating intestinal inflammation [31]. Immunohistochemical analysis confirmed that DSS markedly reduced ZO-1/occludin expression while elevating TNF-α levels. Critically, DOP intervention reversed these pathological alterations, demonstrating its therapeutic capacity to attenuate intestinal inflammation through restoring the intestinal barrier. However, our results indicated that both goblet cell density and tight junction protein occludin expression in DOPJ-treated groups failed to exhibit dose-dependent increases. This non-monotonic response may be attributable to underlying molecular mechanisms. We hypothesize that DOP polysaccharides engage specific cell surface receptors (e.g., TLR4, Dectin-1), wherein low doses sufficiently activate barrier-repair pathways, while higher doses provoke receptor overstimulation, causing either (1) downstream signal desensitization or (2) induction of negative feedback loops that counteract beneficial effects. In addition, this high-viscosity dietary fibers physically impede nutrient assimilation kinetics—delaying gastric emptying, slowing intestinal transit, and restricting nutrient diffusion. These properties may also alter microbial growth dynamics in the inflamed colon, suggesting that the intrinsic viscosity of high-dose, poorly degraded DOP polymers could counteract intestinal mucosa repair mechanisms [32]. The above discussion requires further studies to delineate the precise mechanisms behind this dose-dependent effect.

16S rRNA sequencing analysis revealed that the composition of the gut microbiota in DOP-intervened mice was significantly altered compared to the DSS model group. Specifically, the DOP intervention groups showed an increase in gut microbial diversity (Figure 3A) and distinct separation in microbial community structure among the different intervention groups (Figure 3B,D). In terms of taxonomic composition, *Candidatus_Saccharimonas* was identified as a potentially beneficial bacterium that may contribute to the prevention or alleviation of colitis [33], whereas *Muribaculaceae* has been reported as a conditional pathogen associated with ulcerative colitis (UC), potentially exacerbating the inflammatory process [34]. Furthermore, *Lachnoclostridium*, which produces short-chain fatty acids (such as butyrate), plays a critical role in maintaining gut health [35], and *Turicibacter* is recognized as a taxon with anti-inflammatory potential [36]. DOP intervention also increased the relative abundance of *Bacillaceae*, an increase that is closely associated with enhanced intestinal barrier function [37]. Collectively, these results demonstrate that DOP, through preventive intervention, effectively modulates the gut microbiota structure in mice with DSS-induced chronic colitis. The mechanism may be related to promoting the proliferation of beneficial bacteria and suppressing the growth of conditional pathogens, thereby contributing to the mitigation of colitis progression.

In addition to its modulating effect on gut microbiota, we further investigated whether DOP induced deeper reprogramming at the host metabolic level. The results suggest that The potential mechanisms underlying the amelioration of chronic colitis by DOP likely encompass a synergistic interplay among multiple metabolic pathways, rather than the regulation of a single pathway.

Niacin and niacinamide metabolism are closely associated with the synthesis of NAD+ and NADP+, because these cofactors regulate inflammatory responses [38]. This suggests that DOP alleviates chronic colitis pathology through anti-inflammatory mechanisms. Additionally, pyrimidine, a fundamental component of nucleic acids (DNA and RNA), may be modulated by DOP to regulate the activity of immune cells (e.g., macrophages, T-cells) [39], potentially enhancing the repair processes of chronic colitis. Nucleotide metabolism, which forms the basis of DNA and RNA synthesis, is intrinsically linked to cell proliferation, repair, and apoptosis [40]. DOP may promote intestinal epithelial cell proliferation and repair by regulating nucleotide metabolism. Cofactors, which are non-protein components essential for enzymatic reactions, are critical for maintaining enzyme activity and physiological function [41]. By modulating cofactor biosynthesis, DOP likely regulates chronic colitis-associated enzymatic pathways, thereby supporting intestinal health. Moreover, DOP modulates α-linolenic acid metabolism, a polyunsaturated fatty acid critical for gut health [42], which may promote intestinal mucosal repair and protection. The above are the common metabolic pathways of DOPY and DOPJ, which likely constitute shared mechanisms for *Dendrobium officinale* polysaccharides in combating intestinal inflammation.

Notably, DOPY and DOPJ exhibit distinct metabolic pathways underlying their biological functions. Unsaturated fatty acids, key regulators of membrane stability and fluidity [43], may mediate DOPY’s beneficial effects on intestinal health through regulation of biosynthesis. DOPY’s antioxidant properties may involve ubiquinone (UQ) biosynthesis, as UQ is a vital antioxidant and energy metabolism cofactor [44]. Furthermore, DOPY regulates folate metabolism, a pathway indispensable for DNA synthesis and repair [45], suggesting its role in enhancing intestinal cell maintenance. However, direct associations between chronic colitis and pathways such as cutin, suberin, wax biosynthesis, and porphyrin metabolism remain less evident.

DOPJ enhances pentose phosphate pathway activity, a major source of NADPH for antioxidant defense and nucleotide synthesis [46], thereby elevating cellular antioxidant capacity and nucleotide production while mitigating chronic colitis-induced oxidative stress. DOPJ also modulates D-amino acid metabolism, a pathway linked to immune signaling [47], potentially attenuating intestinal inflammation through immune cell regulation. Additionally, DOPJ promotes nucleotide sugar biosynthesis, a process essential for cellular regeneration [48], supporting intestinal epithelial recovery.

These findings indicated that beyond common pathways, DOPY regulated chronic colitis-related metabolism through membrane stability/fluidity, UQ biosynthesis, and DNA synthesis/repair. DOPJ alleviated chronic colitis by modulating antioxidant responses, nucleotide synthesis, immune cell activity, and cellular regeneration. Such divergences may represent key determinants of differential bioactivity among *Dendrobium officinale* polysaccharide fractions.

This study positions DOP as a potential natural intervention strategy, offering an alternative approach to the prevention and management of chronic colitis that differs from conventional pharmaceutical treatments. As a functional food component, DOP exhibits low toxicity and minimal side effects, making it suitable for daily dietary supplementation aimed at long-term maintenance of intestinal health and prevention of colitis risk. The potent protective effects of DOP observed here, particularly its ability to modulate gut microbiota, strengthen the intestinal barrier, and suppress systemic inflammation, are mechanisms that are also highly relevant to the treatment of established colitis. For instance, the restoration of a healthy gut microbiome and the enhancement of mucosal healing are primary goals in managing IBD patients. Our data suggest that DOP, by simultaneously targeting these multiple key pathways, holds promise as a therapeutic agent.

However, this study has several limitations. First, there are species differences in pathological mechanisms between the DSS-induced chronic colitis mouse model and human disease; thus, further clinical trials are required to validate the efficacy and safe dosage of DOP in humans. Second, the experimental design focused on preventive intervention during disease initiation and did not investigate the therapeutic effect of DOP on established colitis, which somewhat limits a comprehensive understanding of its full therapeutic potential. To bridge this gap, future clinical studies should be designed where DOP is administered to patients with active UC. Initially, this could be explored as an adjunctive therapy alongside conventional first-line treatments (e.g., mesalazine), potentially to enhance efficacy or reduce the dosage of conventional drugs and their side effects. The optimal clinical protocol (e.g., dosage, formulation, and duration of treatment) needs to be determined in phased clinical trials, starting from our effective dose in this preclinical study.

## 5. Conclusions

In summary, the present study systematically elucidated the potential mechanism of DOP intervention in the treatment of chronic colitis by integrating pharmacodynamic, metabolomic, and microbiomic analysis. The results showed that DOP ameliorated chronic colitis symptoms in mice by preserving intestinal barrier integrity, modulating intestinal microbiota composition, and synergistically targeting multiple metabolic pathways. This study sheds light on the positive impact of DOP on reshaping gut microbiota and protecting gut barrier function, providing valuable insights for the potential development and application of DOP.

## Data Availability

The original contributions presented in this study are included in the article/Supplementary Materials. Further inquiries can be directed to the corresponding author.

## Supplementary Materials

The following supporting information can be downloaded at: https://www.mdpi.com/article/10.3390/metabo15110708/s1. Figure S1: Model verification diagram; Table S1: Scoring system for DAI; Table S2: Information of DOPY potential differential metabolites; Table S3: Information of DOPJ potential differential metabolites.

## Abbreviations

The following abbreviations are used in this manuscript:

DOP	*Dendrobium officinale* polysaccharides
DSS	Dextran sulfate sodium
DAI	Disease Activity Index
TNF-α	Tumor Necrosis Factor-alpha
ZO-1	Zonula occludens-1
DO	*Dendrobium officinale*
DOPY	polysaccharides from *Dendrobium officinale* leaves
DOPJ	polysaccharides from *Dendrobium officinale* stems
HPGPC	High-performance gel permeation chromatography
FT-IR	Fourier Transform Infrared Spectroscopy
SASP	Sulfasalazine
DOPY-L	low-dose polysaccharides from *Dendrobium officinale* leaves
DOPY-H	high-dose polysaccharides from *Dendrobium officinale* leaves
DOPJ-L	low-dose polysaccharides from *Dendrobium officinale* stems
DOPJ-H	high-dose polysaccharides from *Dendrobium officinale* stems
H&E	Hematoxylin and Eosin
PBS	Phosphate-buffered saline
IL-6	Interleukin-6
IL-10	Interleukin-10
ELISA	Enzyme-linked immunosorbent assay
ANOVA	Analysis of variance
UC	Ulcerative colitis
UQ	Ubiquinone
