# Supplementary material for: Dendrobium officinale Polysaccharide Relieves the DSS-Induced Chronic Colitis in C57BL/6J Mice and Regulates Colonic Microflora Structure
Source: Metabolites. 2025 Oct 30;15(11):708. doi: 10.3390/metabo15110708 (PMC12654858; doi:10.3390/metabo15110708)
Supplement: Supplementary file 1 [file metabolites-15-00708-s001.zip › metabolites-3882079-supplementary.pdf]

Supplementary Material

Metabolomic analysis

The LC-MS analysis was performed on a Thermo Scientific™ UHPLC-Q Exactive™ HF-X system equipped with ultra-high performance liquid chromatography and Fourier transform mass spectrometry. Chromatographic separation was achieved using an ACQUITY UPLC HSS T3 column (100 mm × 2.1 mm i.d., 1.8 μm; Waters, Milford, USA) with a 3 μL injection volume. The mobile phase consisted of solvent A (Water-acetonitrile (95:5, v/v) with 0.1% formic acid) and solvent B (Acetonitrile-isopropanol-water (47.5:47.5:5, v/v/v) with 0.1% formic acid). For positive ion mode, the elution protocol comprised: (1) 0-3 min: 0% B (isocratic), (2) 3-4.5 min: 0→35% B (linear gradient), (3) 5.0-6.3 min: instant 100% B (step gradient), and (4) 6.4-8.0 min: 0% B (re-equilibration). Negative ion mode employed: (1) 0-1.5 min: 0% B (isocratic), (2) 1.5-4.5 min: 0→30% B (multi-linear gradient: 1.5 min 5%, 2.0 min 10%), (3) 5.0-6.3 min: 100% B (step gradient), and (4) 6.4-8.0 min: 0% B (system re-equilibration).

Mass spectrometry analysis was conducted in both positive and negative ionization modes with a full-scan mass range of *m/z* 70–1050. Instrument parameters included a sheath gas flow of 50 arb, auxiliary gas flow of 13 arb at 425°C, spray voltages of +3,500 V (positive) and -3,500 V (negative), an ion transfer tube maintained at 325°C, and stepped normalized collision energies (NCE) of 20, 40, and 60 eV. Full MS spectra were acquired at 60,000 resolution (FWHM at *m/z* 200) while data-dependent MS/MS scans operated at 7,500 resolution using dynamic exclusion during

data-dependent acquisition (DDA) mode.

Supplementary Figure

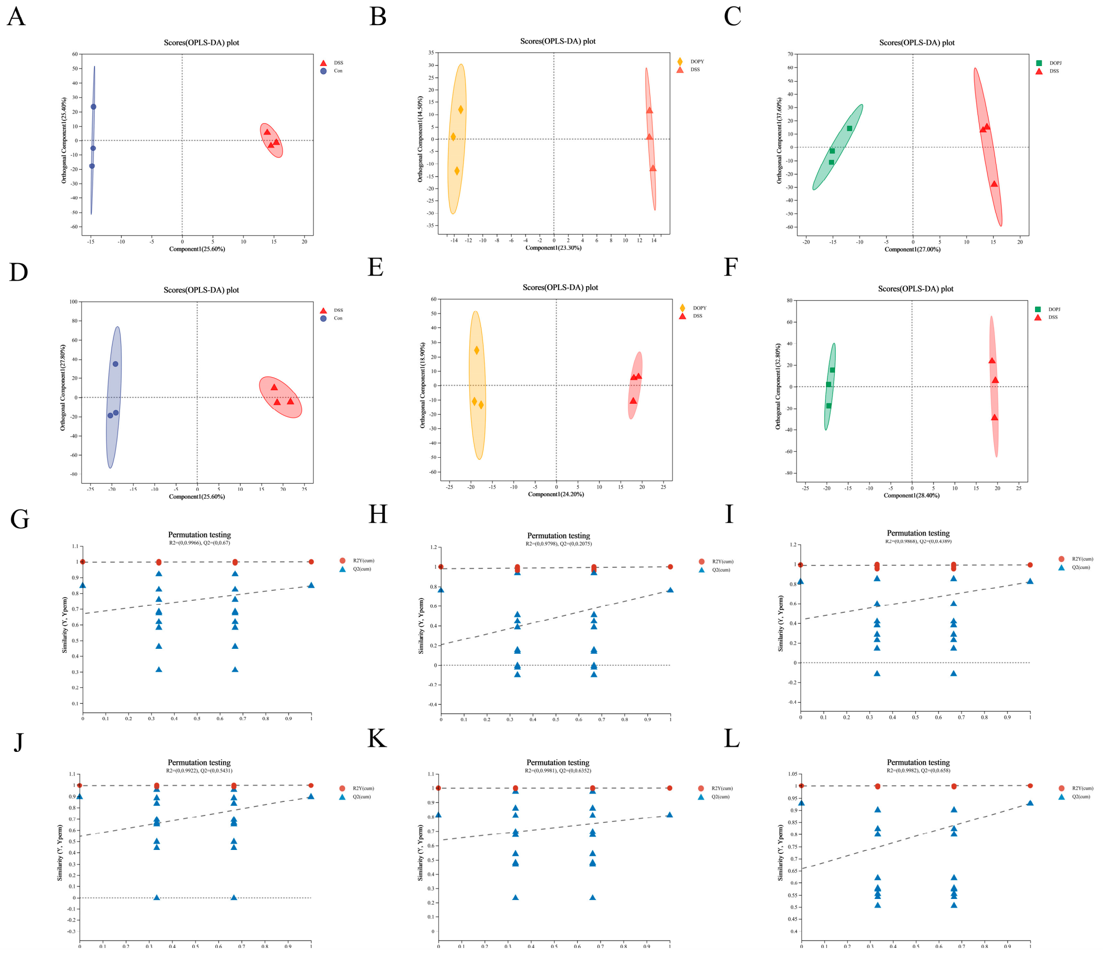

Figure S1. Model verification diagram. Orthogonal least partial squares discriminant analysis (OPLS-DA) of Con group vs DSS group (A), DSS group vs DOPY group (B), and DSS group vs DOPJ group (C) in positive ionization mode. OPLS-DA of Con group vs DSS group (D), DSS group vs DOPY group (E), and DSS group vs DOPJ group (F) in negative ionization mode. OPLS-DA substitution test analysis of Con group vs DSS group (G), DSS group vs DOPY group (H), and DSS group vs DOPJ group (I) in positive ionization mode. OPLS-DA substitution test analysis of Con group vs DSS group (J), DSS group vs DOPY group (K), and DSS group vs DOPJ group (L) in negative ionization mode. Con: normal control group; DSS: model control group; DOPY: 50 mg/kg DOPY group; DOPJ: 100 mg/kg DOPJ group.

Table S1 Scoring system for DAI

Score	Weight loss	Feces appearance	Blood in feces
0	0%	Normal	No blood
1	1-5%	Loose	Observed blood
2	5-10%	Watery diarrhea	
3	10-20%	Slimy diarrhea, little blood	Gross bleeding
4	>20%	Severe watery diarrhea with blood	

Table S2 Information of DOPY potential differential metabolites.

Metabolite	Molecular Formula	VIP	Trends	
			Con vs DSS	DSS vs DOPY
Nicotinic acid ribonucleoside	C11H14NO6+	4.0138	↑	↓
Diflunisal	C13H8F2O3	3.3239	↑	↓
Ara-HX	C10H12N4O5	3.3239	↑	↓
2'-Deoxyadenosine 5'-phosphate	C10H14N5O6P	2.8994	↑	↓
3,4-Dihydroxyphenylpropanoate	C9H10O4	2.8965	↑	↓
4-Oxododecanedioic Acid	C12H20O5	2.8196	↑	↓
Inosine	C10H12N4O5	2.7172	↑	↓
5'-Thymidylic Acid	C10H15N2O8P	2.482	↑	↓
Chakanoside I	C14H18O7	2.4539	↑	↓
Senkyunolide N	C12H18O4	2.4142	↑	↓
Spongothymidine	C10H14N2O6	2.3549	↑	↓
Deoxycytidylic acid	C9H14N3O7P	2.3549	↑	↓
Sclareol	C20H36O2	2.2447	↑	↓
Morroniside	C17H26O11	2.2223	↑	↓
N(epsilon)-(Carboxymethyl)hydroxylysine	C8H16N2O5	2.1751	↓	↑
Porphobilinogen	C10H14N2O4	2.1214	↑	↓
Biopterin	C9H11N5O3	2.0802	↓	↑
Guanosine	C10H13N5O5	1.9821	↑	↓
Arctinol A	C12H10OS2	1.9263	↑	↓
N-(Carbomethoxyacetyl)-4-S-chlorotryptophan	C15H15ClN2O5	1.8744	↑	↓
Vulgarole	C12H20O3	1.859	↓	↑
N-(2-hydroxy-3-methoxy-2-methylpropyl)pyridine-2-carboxamide	C11H16N2O3	1.7982	↑	↓
Islatravir	C12H12FN5O3	1.639	↓	↑
7,4'-Dihydroxyflavone	C15H10O4	1.5309	↑	↓
Cis-9,10-Epoxystearic acid	C18H34O3	1.5023	↑	↓

Tuberose lactone	C12H18O2	1.4952	↓	↑
Linolenic Acid	C18H30O2	1.4859	↑	↓
Octaethyleneglycol monododecyl ether	C28H58O9	1.4264	↑	↓
(3R)-3,4-Dihydroxy-3-(hydroxymethyl)butanenitrile 4-glucoside	C11H19NO8	1.332	↑	↓
Tyrosylhydroxyproline	C14H18N2O5	1.2984	↓	↑
3-Hydroxypropyl methacrylate	C7H12O3	1.2838	↑	↓
Manoyl oxide	C20H34O	1.2095	↑	↓
Citrazinic Acid	C6H5NO4	1.1366	↑	↓
Paclobutrazol	C15H20ClN3O	1.1213	↓	↑
Methionyl-Histidine	C11H18N4O3S	1.102	↓	↑
Demethylphylloquinone	C30H44O2	1.0886	↑	↓
3,4,5-Trihydroxypentanoylcarnitine	C12H23NO7	1.0348	↓	↑

Table S3 Information of DOPJ potential differential metabolites.

Metabolite	Molecular Formula	VIP	Trends	
			Con vs DSS	DSS vs DOPJ
Arginylglutamine	C11H22N6O4	5.533	↓	↑
Ara-HX	C10H12N4O5	4.0947	↑	↓
13-Nor-6-eremophilene-8,11-dione	C14H20O2	3.9187	↓	↑
Nicotinic acid ribonucleoside	C11H14NO6+	3.7677	↑	↓
D-Sedoheptulose 7-phosphate	C7H15O10P	3.5682	↑	↓
Diflunisal	C13H8F2O3	3.5131	↑	↓
Neocnidolide	C12H18O2	3.4791	↓	↑
Arginyl-Gamma-glutamate	C11H22N6O4	3.442	↓	↑
Inosine	C10H12N4O5	3.4177	↑	↓
3'-Adenylic Acid	C10H14N5O7P	3.2996	↑	↓
Artemin	C15H22O4	3.2569	↓	↑
2'-Deoxyadenosine 5'-phosphate	C10H14N5O6P	3.062	↑	↓
4-Oxododecanedioic Acid	C12H20O5	2.7976	↑	↓
3,4-Dihydroxyphenylpropanoate	C9H10O4	2.7959	↑	↓
Biopterin	C9H11N5O3	2.7553	↑	↓
Guanosine	C10H13N5O5	2.6015	↑	↓
5'-Thymidylic Acid	C10H15N2O8P	2.5855	↑	↓
Deoxycytidylic acid	C9H14N3O7P	2.5808	↑	↓
Trinexapac-ethyl	C13H16O5	2.4918	↓	↑
Senkyunolide N	C12H18O4	2.4186	↓	↓
Chakanoside I	C14H18O7	2.4097	↑	↓
Islatravir	C12H12FN5O3	2.4033	↓	↑
N(epsilon)-(Carboxymethyl)hydroxylysine	C8H16N2O5	2.3861	↑	↓
6-Hydroxyindoxyl sulfate	C9H9NO4S	2.386	↓	↑
Tryptophyl-Glutamine	C16H20N4O4	2.3646	↓	↑
Filipin II	C35H58O10	2.3622	↓	↑
8-Deoxy-11,13-dihydroxygrosheimin	C15H20O5	2.3015	↑	↓
N-(2-hydroxy-3-methoxy-2-	C11H16N2O3	2.2071	↓	↑

methylpropyl)pyridine-2-carboxamide				
2,6-Pyridinedicarboxylic Acid	C7H5NO4	2.1275	↑	↓
(3E,5E,8Z)-Deca-3,5,8-trienedioylcarnitine	C17H25NO6	2.1163	↑	↓
N(4)-Methylcytosine	C5H7N3O	2.0722	↑	↓
Arctinol A	C12H10OS2	2.044	↑	↓
Equol 4'-O-glucuronide	C21H22O9	1.7724	↓	↑
Quinolinic Acid	C7H5NO4	1.6952	↑	↓
Tiglic acid	C5H8O2	1.6497	↓	↑
Artabsin	C15H20O3	1.6438	↓	↑
Cinncassiol C3	C20H30O7	1.5856	↑	↓
Tuliposide A	C11H18O8	1.4817	↑	↓
Gibberellin A53	C20H28O5	1.4724	↓	↑
Vulgarole	C12H20O3	1.4536	↑	↓
5-Amino-2-oxopentanoic acid	C5H9NO3	1.4094	↓	↑
7,4'-Dihydroxyflavone	C15H10O4	1.4094	↑	↓
(3beta,5alpha,6alpha,7alpha,22E,24R)-5,6-Epoxyergosta-8,14,22-triene-3,7-diol	C28H42O3	1.3203	↓	↑
Prolylproline	C10H16N2O3	1.2891	↓	↑
Queuine	C12H15N5O3	1.2878	↓	↑
(6E,8R,10Z)-8-hydroxy-3-oxohexadecadienoic acid	C16H26O4	1.2743	↓	↑
(3Z,6Z)-3,6-Nonadienal	C9H14O	1.2071	↓	↑
Methionyl-Histidine	C11H18N4O3S	1.2006	↓	↑
16-alpha-Hydroxyandrosterone	C19H30O3	1.2001	↓	↑
Octaethyleneglycol monododecyl ether	C28H58O9	1.1992	↑	↓
Beta-D-ribosylnicotinate	C11H13NO6	1.1882	↓	↑
12-oxo-20-dihydroxy-leukotriene B4	C20H30O6	1.1114	↓	↑
4(1H)-Pyridinone, 2-ethyl-3-hydroxy-1-(2-hydroxyethyl)-	C9H13NO3	1.0944	↓	↑
Dimethyl (R)-pyrrolidine-1,2-	C8H13NO4	1.0734	↓	↑

dicarboxylate				
Lauryldiethanolamine	C16H35NO2	1.0594	↑	↓
10-Hydroxy-2-oxabicyclo[6.2.2]dodeca-1(10),8,11-trien-3-one	C11H12O3	1.0317	↓	↑
